# Botulinum Toxin-A Injection in Chronic Pelvic Pain Syndrome Treatment: A Systematic Review and Pooled Meta-Analysis

**DOI:** 10.3390/toxins14010025

**Published:** 2022-01-01

**Authors:** Andrea Panunzio, Alessandro Tafuri, Giovanni Mazzucato, Clara Cerrato, Rossella Orlando, Vincenzo Pagliarulo, Alessandro Antonelli, Maria Angela Cerruto

**Affiliations:** 1 Department of Urology, Azienda Ospedaliera Universitaria Integrata Verona, University of Verona, Piazzale Aristide Stefani 1, 37126 Verona, Italy; panunzioandrea@virgilio.it (A.P.); dott.giovannimazzucato@gmail.com (G.M.); clara.cerrato01@gmail.com (C.C.); orlandorossella92@gmail.com (R.O.); alessandro_antonelli@me.com (A.A.); 2 Department of Urology, “Vito Fazzi” Hospital, 73100 Lecce, Italy; enzopagliarulo@yahoo.com

**Keywords:** chronic pelvic pain, botulinum toxin A, bladder pain syndrome, prostate pain syndrome, scrotal pain, myofascial pain, gynecological pelvic pain

## Abstract

Introduction: Pain management of patients with chronic pelvic pain syndrome (CPPS) is challenging, because pain is often refractory to conventional treatments. Botulinum toxin A (BTX-A) may represent a promising therapeutic strategy for these patients. The aim of this systematic review was to investigate the role of BTX-A in CPPS treatment. Methods: We reviewed the literature for prospective studies evaluating the use of BTX-A in the treatment of CPPS. A comprehensive search in the PubMed, Scopus, Web of Science, and Cochrane Central Register of Controlled Trials databases was performed from English language articles published between January 2000 and October 2021. The primary outcome was to evaluate pain improvement in CPPS after BTX-A treatment. Pooled meta-analysis of the included studies, considering the effect of BTX-A on pain evaluated at last available follow-up compared to baseline values, was performed together with meta-regression analysis. Results: After screening 1001 records, 18 full-text manuscripts were selected, comprising 13 randomized clinical trials and five comparative studies. They covered overall 896 patients of both sexes and several subtype of CPPS (interstitial cystitis/bladder pain syndrome, chronic prostatitis/prostate pain syndrome, chronic scrotal pain, gynecological pelvic pain, myofascial pelvic pain). The clinical and methodological heterogeneity of studies included makes it difficult to do an overall estimation of the real effect of BTX-A on pain and other functional outcomes of various CPPS subtypes. However, considering pooled meta-analysis results, a benefit in pain relief was showed for BTX-A-treated patients both in the overall studies populations and in the overall cohorts of patients with CPP due to bladder, prostate, and gynecological origin. Conclusions: BTX-A could be an efficacious treatment for some specific CPPS subtypes. Higher level studies are needed to assess the efficacy and safety of BTX-A and provide objective indications for its use in CPPS management.

## 1. Introduction

Chronic pelvic pain (CPP) is defined as a chronic or persistent pain perceived in structures related to the pelvis of either men or women, lasting for at least six months. It is often associated with negative cognitive, behavioral, sexual, and emotional consequences as well as with symptoms suggestive of lower urinary tract, sexual, bowel, pelvic floor or gynecological dysfunction. Causes may include local pathology such as infections, malignancy or primary anatomical, functional or neurogenic disease of the pelvic organs. Otherwise, when there is no evidence of proven underlying disease accounting for the pain, it refers to a chronic pelvic pain syndrome (CPPS) [1].

The etiology of CPP is difficult to determine, as the physiopathology is complex and may vary between patients’ population and disease subtype. Several theories and associated findings are currently under investigations [2]. Both animal and clinical studies have historically evaluated the physiopathology of this syndrome proposing mechanisms in which pain is maintaining even in the absence of peripheral stimuli or a recognized pathology [3]. Moreover, it is difficult to assess the real prevalence of CPPS, considering both sexes and all types of subclassification, mainly because of the different diagnostic criteria and the overlapping symptoms with other diseases. A few studies have tried to investigate the prevalence of CPP, and with different results. Mathias et al. reported a prevalence of CPP among US women aged 18–50 years around 14.7%, and in more than half of cases the etiology was unknown [4]. In a more recently study, Marszalek et al. described a prevalence of symptoms suggestive of CPP of 5.7% and 2.7% in women and men, respectively [5].

The management of CPPS is based on a bio-psychosocial model which includes an active patients’ involvement. Pharmacological and non-pharmacological interventions such as psychotherapy, physiotherapy, drugs and invasive treatments rarely works in isolation and often need to be considered together as a part of a personalized treatment strategy [1,6].

Botulinum toxin is currently used for the treatment of various pain disorders. It is a neurotoxin well known due of its action that blocks the exocytosis of the acetylcholine on the presynaptic cholinergic peripheral nerves. This ends in a skeletal muscle relaxation and in an analgesic effect. There are seven different types of botulinum neurotoxins, named from A to G [7]. The serotype A (BTX-A) was first investigated in 1990 for the treatment of detrusor external sphincter dyssynergia in patients with spinal cord injuries [8]. Few years later it was used against neurogenic detrusor overactivity. Nowadays neurotoxin A is also used as a third line treatment against non-neurogenic overactive bladder [9]. International guidelines already considered BTX-A, for the management of chronic prostatitis (CP)/prostate pain syndrome (PPS) and functional anorectal pain [1]. However, there are conflicting results in literature, among studies assessing the real effect of BTX-A in the treatment of CPP. Aim of this systematic review is to investigate the efficacy of BTX-A injection in CPPS management.

## 2. Results

**Figure 1:**
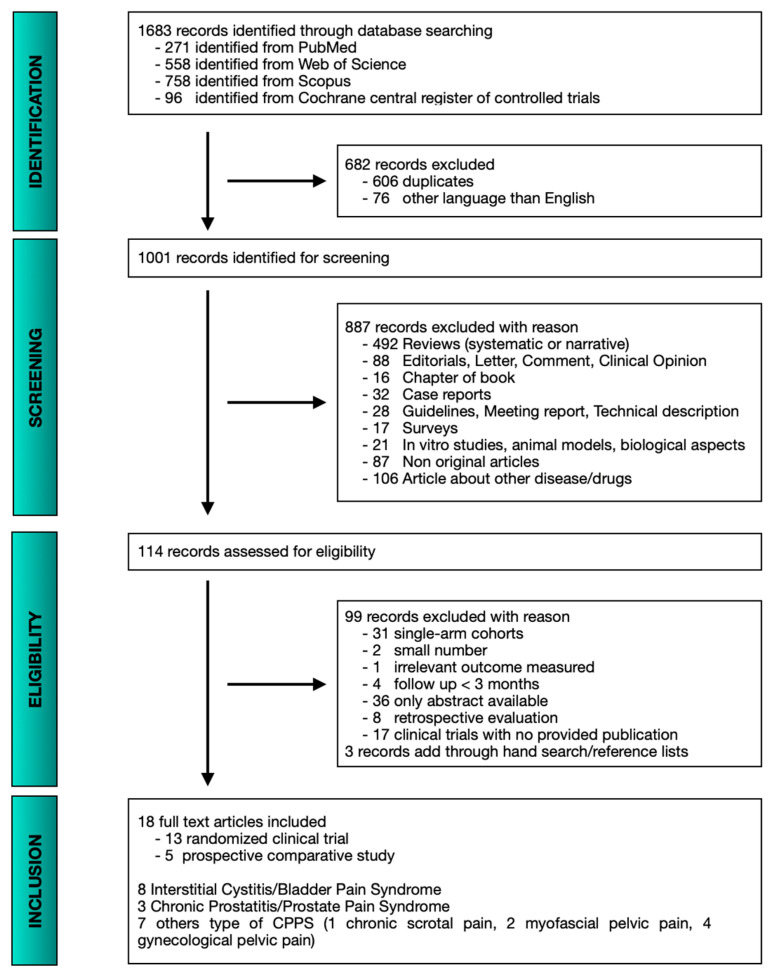
PRISMA flow diagram for systematic review assessing the efficacy of BTX-A in the treatment of CPPS.

The PRISMA diagram shows the literature research results (Figure 1). We identified 1001 records overall for screening. A total of 114 records were retrieved and assessed for eligibility. Finally, 18 full-text manuscripts, including 13 randomized clinical trials (RCTs) and five prospective comparative studies met inclusion criteria and were included. Regarding urological field, eight studies assessed the use of BTX-A in bladder pain syndrome (BPS)/painful bladder syndrome (PBS)/interstitial cystitis (IC), three studies in prostate pain syndrome (PPS)/chronic prostatitis (CP) and one study in chronic scrotal pain (CSP). Moreover, four studies investigate the role of botulinum toxin injections in gynecological pelvic pain (GPP) and two studies evaluated its role in myofascial pelvic pain (MPP). The characteristics of these studies, including patients’ demographics details are summarized in Supplementary Table S1. There was a significant heterogeneity in the design and in the outcomes measured by each study and it means that is difficult to estimate the global response of BTX-A among pain and other functional outcomes. To facilitate data comparison and interpretation, we divided all the studies evaluated into major subtypes of CPPS and reported main data extracted among the reduction in pain score and frequency and nocturia episodes in separate dedicated tables.

### 2.1. Evidence Synthesis

#### 2.1.1. Bladder Pain Syndrome/Interstitial Cystitis

We included eight studies investigating the role of BTX-A in the context of IC/BPS, 5 RCT and three comparative studies [10,11,12,13,14,15,16,17] (Supplementary Table S2). Overall participants included 319 patients. The type and dose of BTX-A administered were variable, with three studies using onabotulinum toxin A (onaBTX-A), one using abobotulinum toxin A (aboBTX-A) and the others not specifying this data, as well as the number and the location of injections, which ranged from 10 to 40, and were carried out in the entire bladder, only in the trigone, or in the bladder body. All manuscripts except one reported that patients before to study participation failed previous conservative treatment and had symptoms of IC/BPS lasting more than six months. The intervention and control groups differed greatly among the studies included. One study compared intradetrusorial BTX-A injections and hydrodistension (HD) with placebo (normal saline injections) and HD [10], one compared BTX-A immediate versus delayed (after one month) injections [11], and two evaluated the differences between sub-urothelial BTX-A injections using a ‘trigonal template’ against a ‘bladder-body/trigone sparing template’ [12,13]. Another RCT compared intradetrusorial BTX-A injections with intravesical instillation of Bacille Calmette-Guerin (BCG) [14]. Finally, two comparative studies investigated the differences in response of BTX-A injections in ulcerative and non-ulcerative IC phenotype [15,16], while one assessed the differences between different doses of BTX-A (100 vs. 200 IU) administered [17]. Five studies used the visual analogical scale (VAS) for pain evaluation ranged from 0 up to 10, two reported the pain experience of patients using the O’Leary Sant questionnaire (OLS), while one used the Likert scale ranged from 0 up to 9. All studies analyzed changes in lower urinary tract symptoms, as the reduction in frequency and nocturia episodes. 

Results hugely differs among studies evaluated. Manning et al. reported that BTX-A injections were associated with no overall improvement in total OLS score, although a benefit was noted in BTX-A patients who showed an improvement of the OLS-PI (problem index) questionnaire at three months [10]. Other two studies showed a significant benefit in favor of BTX-A as compared to the control arm. Specifically, El-Bahnasy et al. reported an improvement in the domains of daytime frequency, nocturia and pelvic pain among women randomized to treatment with BTX-A [14]. Kuo et al. showed that although IC/BPS symptoms score significantly decreased in all the three arms of the study (BTX-A 200 IU plus HD vs. BTX-A 100 IU vs. HD), VAS score reduction was significant only in the BTX-A group after three or in some cases after six months of follow up [17]. Two manuscripts analyzed differences in response among BTX-A administration only in the trigone or outside of trigone or in the entire bladder, showing that there was no location dependent improvement in IC/BPS symptom scores. Specifically, Evans et al. reported that patients in both groups experienced significant improvement in OLS and pain/urgency/frequency (PUF) questionnaire, both at 30 and 90 days, regardless of which injection template was used [12]. Similarly, Jiang et al. demonstrated an improvement in VAS and OLS after treatment, even though no changes was noted among urinary frequency and urodynamic parameters from baseline to eight weeks between two groups [13]. Finally, two studied experienced opposite results among the response of BTX-A in ulcerative and non-ulcerative IC. Lee et al. showed that repeated intradetrusorial BTX-A injections provide effective outcomes in 50% of patients with non-ulcer IC/BPS but did not benefit any patient with ulcer type [15]. In the study of Pinto et al., both groups had comparable clinical response to intra-trigonal BTX-A injections with improvement in pain, frequency and nocturia, suggesting that maybe pain was not directly associated with IC/BPS phenotype [16].

#### 2.1.2. Prostate Pain Syndrome/Chronic Prostatitis

Three studies, including two RCT and one prospective comparative study, evaluated the role of BTX-A for CP/PPS treatment (Supplementary Table S3) [18,19,20]. Overall, 180 men, with symptoms refractory to antibiotics, alpha blockers or anti-inflammatory agents, were included. All of three studies used onaBTX-A delivered in three or four sites bilaterally in the prostate using the transurethral or the transrectal approach and assessed the response in pain before and after treatment using the VAS (0–10) scale and/or the National Institutes of Health Chronic Prostatitis Symptoms Index (NIH-CPSI) ranged from 0 up to 21.

Two studies compared transurethral intraprostatic BTX-A injections with cystoscopy alone [18] or normal saline injections [19], while one RCT investigated differences between transurethral and transrectal intraprostatic injections [20]. All papers demonstrated that BTX-A intraprostatic injections may be an effective and safety therapeutic option to ameliorate CP/PPS symptoms, especially pain. Abdel Meguid et al. showed that baseline NIH-CPSI and VAS scores in treated arm decreased by 68.2% and 79% at three months, respectively (*p* < 0.0001); instead, none of control patients demonstrated significant scores changes from baseline [18]. Even the results of Falahatkar et al. showed that NIH-CPSI total and subscale score, VAS, QoL scores and frequencies of diurnal and nocturnal urinations significantly improved both at one, three and six months in men underwent BTX-A 100 or 200 IU transurethral intraprostatic injections (*p* < 0.05); in contrast, none of these values showed improvement in placebo cohort [19]. Finally, the study of El Enen et al. showed that BTX-A was more effective in patients with small prostate and short symptoms duration and that the transrectal approach provided better result than the transurethral one [20].

#### 2.1.3. Chronic Scrotal Pain

One single RCT assessed the efficacy of BTX-A injections in CSP (Supplementary Table S4) [21]. This study included 60 men with unilateral or bilateral chronic testicular pain who showed incomplete response to previous treatment with nonsteroidal anti-inflammatories, support, neuropathic medication and antibiotics. It compared BTX-A (200 IU) injection plus local anesthesia (LA) in the spermatic cord against normal saline injection plus LA and showed no superiority of BTX-A for pain control.

#### 2.1.4. Gynecological Pelvic Pain

Four studies including three RCT and one prospective comparative study, with 194 overall female patients, evaluated the benefit of BTX-A in the treatment of GPP (Supplementary Table S5) [22,23,24,25]. All these studies used onaBTX-A at different doses (from 20 to 100 IU) and with different numbers and sites of injections. One study compared the response to single BTX-A injection versus repeated treatment in responders’ patients [23], while the others compared BTX-A with normal saline injections [22,24,25]. In all studies pain assessment before and after treatment was evaluated using the VAS score ranged from 0 up to 10 or to 100. Moreover, sexual function was assessed with dedicated questionnaires as the female sexual function index (FSFI) and the female sexual distress scale (FSDS). Petersen et al. showed that injection of 20 IU of BTX-A in the musculus bulbo-spongiosus of women with vestibulodynia did not reduce pain or resulted in sexual functions and quality of life improvement at six months compared to placebo. Both BTX-A and normal saline groups patients experienced a reduction in pain at six months (*p* < 0.001); however, no significant difference in the median VAS score or in sexual function questionnaire scores was observed between the two groups [22]. Diomande et al. demonstrated no differences between BTX-A subcutaneous (50 or 100 IU) injections in vaginal vestibule compared to placebo in improving symptoms of provoked vestibulodynia, even if all three study arms experienced a reduction in pain score at three months follow up after a single injection [24]. Finally, in the paper of Abbott et al. it was demonstrated that BTX-A may be a useful agent in women with pelvic floor muscle spasm resulting in CPP who did not respond to previous conservative therapy; a significant change from baseline for dyspareunia and non-menstrual pain, both evaluated through the VAS scale, was noted for the BTX-A group (*p* < 0.001 and *p* < 0.009, respectively) [25].

#### 2.1.5. Myofascial Pelvic Pain

Two RCT assessed the used of BTX-A in MPP (Supplementary Table S6) [26,27]. Overall, participants included 25 males and 113 females. One study report that patients had pain refractory to analgesic and physiotherapy, which lasted around five years; for the other one, no data were available for the duration of symptoms and type of therapy tried prior to study participation. Dessie et al. compared onaBTX-A (200 IU) pelvic floor injections with normal saline ones, finding no significant differences at two, four and 12 weeks between the intervention and the placebo arm of the study among change of pain [26]. Otherwise, Lévesque et al. compared incoBTX-A 50 and 100 IU injections in the obturator internus muscle and levator ani muscles, respectively plus LA with LA alone. Even in this study, no significant differences were noted between the two groups at two months; however, both groups showed significant alleviations of global pelvic pain [27]. Results from both studies did not support the use of botulinum toxin A in patients with MPP.

### 2.2. Pooled and Meta-Regression Analysis

**Figure 2:**
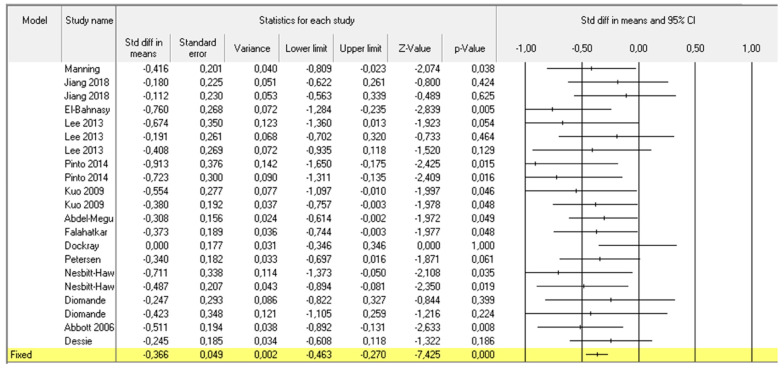
Pooled meta-analysis results and Forrest plot showing the differences in pain scores between baseline and last follow up assessment for 21 included cohorts of 14 considered studies.

**Figure 3:**
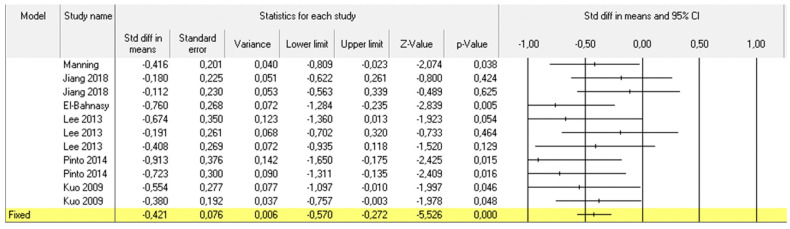
Pooled meta-analysis results and Forrest plot showing the differences in pain scores between baseline and last follow up assessment for 11 included cohorts of six considered studies that evaluated the efficacy of BTX-A injection for IC/BPS.

**Figure 4:**
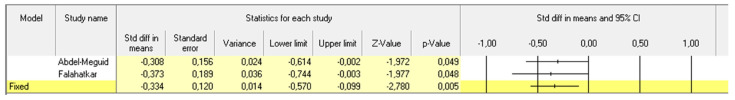
Pooled meta-analysis results and Forrest plot showing the differences in pain scores between baseline and last follow up assessment for 2 included cohorts of two considered studies that evaluated the efficacy of BTX-A injection for CP/PPS.

**Figure 5:**
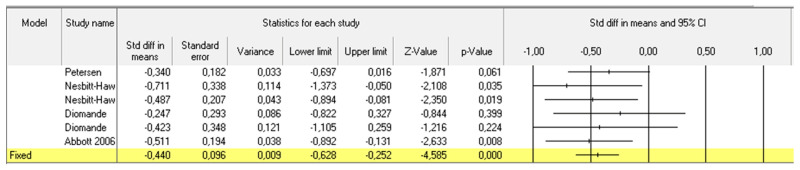
Pooled meta-analysis results and Forrest plot showing the differences in pain scores between baseline and last follow up assessment for 6 included cohorts of four considered studies that evaluated the efficacy of BTX-A injection for GPP.

For the pooled meta-analysis we included 21 cohorts of BTX-A-treated patients coming from 14 studies [10,13,14,15,16,17,18,19,21,22,23,24,25,26] for a total of 447 patients. Funnel plot based on standard error by Hedges’ among selected cohorts showed a low heterogeneity between the study (Figure S1). The difference between pain scores at baseline and at last available follow up was considered. When we assessed all patients that underwent BTX-A treatment according to the available data, a significant improvement in pain perception, related to the scale adopted, was showed in the overall cohort (Figure 2). When we considered overall treated populations grouped according to CPPS subtypes, we found a significant improvement in pain relief in IC/PBS (192 patients; Figure 3), CP/PPS (73 patients; Figure 4) and GPP (120 patients; Figure 5). 

Moderator meta-regression analysis did not find any significant influence of the considered factors. However, the number of site injections showed a significant trend in IC/BPS patients that underwent only trigonal BTX-A injections (Supplementary Tables S7–S12).

## 3. Discussion

Management of CPPS needs a holistic approach with active patient involvement. Indeed, most of the time single interventions do not work in isolation and should be considered together in order to give the patients a personalized care. Treatments may include psychological therapy, due to the presence of associated negative cognitive symptoms such as anxiety, and behavioral consequences, physical therapy, pharmaco-therapy and sometimes surgery. CPPS should be addressed in a multispecialty and multidisciplinary environment with the collaboration of different experts, such as urologists, gynecologists, pain therapists and physiotherapists, to take in consideration all patients symptoms. History, followed by physical examination, are the first steps to evaluate patients with CPP and collect all functional and pain-related symptoms aiming to identify the CPPS subtype and select the best treatment options [1].

Botulinum is a bacterial neurotoxin acting as a presynaptic neuromuscular blocking agent and inhibiting the release of acetylcholine from nerve fibers endings. This effect determines a transitory skeletal muscle relaxation and produces an analgesic effect by reducing muscle hyperactivity [7]. Nociceptive effects of botulinum toxin A are also showed on sensory neurons through the preventing of the neurotransmitter release, from sensory peripheral nerve fibers, involved in pain genesis [28]. Botulinum toxin is a recognized treatment option for autonomic disorders, spasticity and hyperkinetic movement disorders, for treating wrinkles in the cosmesis [29], for upper limb spasticity, hemifacial spasm, blepharospasm and hyperidrosis [30]. It has been also used to treat various pain disease, such as cervical dystonia and chronic migraine, which remains the only approved pain indication, even though several clinical trials have investigated the efficacy and safety of botulinum toxin in other different pain condition [31].

BTX-A for the treatment of CPPS is still in off-label use. In the European Association of Urology (EAU) guidelines, BTX-A is reported as a treatment option only for CP/PPS, pelvic floor pain and chronic primary anal pain syndrome [1]. Several studies have addressed the role of BTX-A in these diseases, even though with no definitive conclusion and subsequentially no absolute indication in favor of its efficacy can be assumed [1].

In this systematic review, we investigated the efficacy of BTX-A injection in CPPS management. We have selected only prospective studies and then have grouped them among CPPS subtypes to facilitate data interpretation, analyses, and comparison. However, the methodologically heterogeneity between the included studies regarding the study design, the definition of CPPS subtype, intervention and control groups, number and sexes of patients, type and dose of drug administered, number and location of injections delivered, outcome measured and time of follow up make it difficult to interpret data uniquely and to draw definitive conclusions.

The studies included in our work used different criteria to define the specific CPPS sub-category. Among IC/BPS five studies used the National Institutes of Diabetes and Digestive and Kidney Disease (NIDDK) criteria, two cystoscopy findings, and one study did not provide any definition. All three studies assessing the role of BTX-A in CP/PPS enrolled men who met the National Institutes of Health criteria for type IIIA /IIIB chronic prostatitis. Patients’ recruitment in the studies investigated the efficacy of BTX-A in GPP or MPP was established according to both clinical findings and diagnostic evaluation.

BTX-A formulation is an important issue to state when making a comparison due to different types of BTX-A showing different potency. Specifically, incobotulinum toxin is reported to be as effective as onabotulinum toxin A, while abobotulinum toxin is less potent compared with the other two formulations [32]. Among selected papers, onaBTX-A was used by twelve studies, one used aboBTX-A, one incoBTX-A, and four did not specify this information. 

It is hard to make a summary on the effect of BTX-A on lower urinary tract symptoms, sexual function and quality of life changes, as well as on pain, comparing baseline and post-treatment times, mainly because the diagnostic tools to evaluate these outcomes used by the included studies are different as well as the time at which the measurements were done. Regarding secondary outcomes addressed by each study, BTX-A reduced frequency and nocturia episodes associated with BPS, while data among sexual and bowel functions were poorly reported in the manuscripts. All the four studies investigating the role of BTX-A in GPP evaluated its benefit on sexual function using several tools, such as VAS scale for dyspareunia, FSFI and FSDS questionnaires and the Marinoff scale, with different results. One showed a significant difference from baseline, for VAS score evaluated dyspareunia both in the BTX-A and control groups [26], while in the study of Diomande et al., results from the Marinoff dyspareunia scale indicated a significant improvement only in patients who underwent BTX-A 50 IU injections in dorsal vestibule, from baseline to three months, compared with the other two arms of the study [24]. Otherwise, Petersen et al. demonstrated no statistically significant improvement of FSFI full score, from baseline until six months of follow up, between the two groups [22]. In addition, Nesbitt et al. showed no statistically significant difference in dysmenorrhea for either group from their baseline scores, while repeated injections of BTX-A can provide benefit for dyspareunia [23].

Finally, another aspect poorly investigated is the real prevalence and nature of adverse events/side effects. As illustrated in the Supplementary Table S13, among IC/BPS studies, the main complications associated with BTX-A injections included urinary tract infections (UTI), which sometimes required the use of antibiotics, transient voiding difficulties, that rarely required catheterization, and gross hematuria. In CP/PPS no serious or systemic complications were recorded, except for mild gross hematuria or hematospermia, while in the GPP groups urinary incontinence, injection site pain and flu-like symptoms were reported. Future studies need to standardize the way to report of the adverse events/side effects and the time of their occurrence.

A recent review by Parsons et al. highlighted the same critical issues of our manuscript. They included 16 studies, most in common with our paper, and considered all CPPS subtypes, even chronic anal pain due to chronic anal fissure, and comprised in the analysis even a comparative retrospective study [33]. They showed that BTX-A reduced pain significantly overall in three studies, two among BPS/IC and one regards CPP/CP, while in none of the other CPPS subtypes studies, patients underwent BTX-A injection in the pelvis, experienced a statistically significant pain relief if compared to the control group [33].

We additionally performed a pooled metanalysis and moderator meta-regression analysis including only BTX-A-treated patients, who were evaluated using the VAS scale, comparing pain at baseline and at last available follow up time. We showed an improvement in pain perception in all cohorts that underwent treatment in the overall population and in specific disease populations, even if some studies did not reach a significant improvement when independently considered. No clinical factors were found to influence the treatment efficacy. However, a trend was found for the number of site injections in IC/BPS patients who underwent BTX-A injection in the bladder trigone only.

## 4. Conclusions

The management of patients with CPPS is challenging, because pain is often refractory to conventional treatments. Botulinum toxin has been used for the treatment of various pain disorders, including CPPS. Actually, in the present systematic review, the methodological heterogeneity of the included studies and the main data reported showed that, even if chronic pelvic pain as well as urinary and sexual symptoms may benefit often from the use of BTX-A injected in pelvic structures, with low rates of complications, the current level of evidence is too low to provide recommendations on its use in daily clinical practice. However, we showed a pooled meta-analysis of prospective studies demonstrating a statistically significant pain relief after BTX-A injection compared to baseline values for CPPS in all evaluated cohorts. To implement information regarding the efficacy, tolerance and safety profile of BTX-A, multicentric RCTs with prospective comparison evaluation and with longer follow up are needed. 

## 5. Methods

### 5.1. Search Strategy

This systematic review was performed following the Preferred Reporting Items for Systematic Review and Meta-Analyses (PRISMA) statement [34]. PubMed, Scopus, Web of Science, and Cochrane Central Register of Controlled Trials databases were searched systematically for English-language articles published from January 2000 up to October 2021 on botulinum toxin injection for the treatment of CPPS. The keywords used for the search were: ‘chronic pelvic pain syndrome’, ‘CPPS’, ‘chronic prostatitis’, ‘prostate pain syndrome’, ‘prostatodynia’, ‘interstitial cystitis’, ‘bladder pain syndrome’, ‘painful bladder syndrome’, ‘genital pain syndrome’, ‘testicular pain’, ‘scrotal pain’, ‘orchialgia’, ‘anal pain syndrome’, ‘levator ani syndrome’, ‘proctalgia’, ‘coccydynia’, ‘irritable bowel syndrome’, ‘botulinum toxin’, and ‘BTX-A’.

### 5.2. Selection of Eligible Studies and Data Extraction

Two paired investigators (A.P. and A.T.) independently screened all titles and abstracts records gathered from literature review to identify potential eligible studies and then evaluated full-text manuscript to determine the final included ones. Any disagreements about eligibility were resolved by discussion between the two investigators until a consensus was reached. We included only RCTs and prospective comparative studies with twenty or more participants, reporting the outcomes of interest and with a minimum of three months follow up. Non-English articles, single-arm studies, retrospective evaluation, meeting abstracts, case reports, editorial commentaries and systematic or narrative reviews were excluded. Reference lists of relevant and recent systematic reviews were manually reviewed to identify supplementary studies of interest. 

The intervention whose efficacy was to be assessed was botulinum toxin type A injection into any pelvic structure to treat CPPS. All CPPS subtypes were included. Control groups could include best clinical practice as suggested by international guidelines, placebo or no treatment. Additional inclusion criteria comprised age of participants > 18 years, presence of patient’s assessment before and after the treatment with botulinum toxin and complete data about outcomes of pain/quality of life, voiding dysfunction and adverse events/side effects.

All data extracted from the included studies were recorded in an electronic database. Collected data included main author and year of publication, country of origin, subtype of CPPS investigated, number, sex and age of participants, dose of BTX-A and number and location of injection administered, duration of symptoms, prior therapies received by patients enrolled in the studies and outcomes measured. The primary outcome was the improvement in pain.

### 5.3. Statistical Analyses

Data were synthesized using meta-analytic methods. For the analysis, only the cohorts of patients thar underwent BTX-A treatment in the considered studies were included if data were available. The analysis was performed comparing baseline pain scores with the last available follow up pain scores. The effect size was calculated using standard mean differences and 95% CI. Data were statistically pooled by the standard meta-analysis approach, meaning that studies were weighted by the inverse of the sampling variance. A test of heterogeneity was applied and the I2 statistic computed. The I2 statistic indicates the proportion of total variation among the effect estimates attributed to heterogeneity rather than sampling error and has the advantage to being intrinsically independent of the number of the studies. Categorical characteristics were treated as moderators and effectiveness was compared across sub-groups formed by these moderators. Continuous characteristics were examined as covariates using random-effects (method of moments) meta-regression. We assessed publication bias using a funnel plot with a significance value on 1-tailed p values. Comprehensive Meta-Analysis V.3 ^©^ software (BIOSTAT, Inc. https://www.meta-analysis.com/ (accessed on 10 November 2021) Comprehensive Meta-Analysis V.2 Software [computer program] was used for statistical analyses.

## Data Availability

All data supporting the reported results are available in the original studies included in the systematic review and were showed in figures and tables included in the present manuscript.

## Supplementary Materials

The following supporting information can be downloaded at: https://www.mdpi.com/article/10.3390/toxins14010025/s1, Table S1. Characteristics of the 18 included studies evaluated the efficacy of BTX-A in CPPS. Table S2. Pain score and Frequency/Nocturia episodes for 8 included studies evaluated the efficacy of BTX-A for IC/BPS. Table S3. Pain score and Frequency/Nocturia episodes for 3 included studies evaluated the efficacy of BTX-A for CP/PPS. Table S4. Pain scores for one included study evaluated the efficacy of BTX-A in CSP. Table S5. Pain scores in 4 included studies evaluated the efficacy of BTX-A in GPP. Table S6. Pain scores in 2 included studies evaluated the efficacy of BTX-A in MPP. Table S7. Meta-regression analysis using concomitant treatment, number of site injections and gender as moderators to test the efficacy of BTX-A injection in pain relief in IC/BPS patients. Table S8. Meta-regression analysis using symptoms duration as moderator to test the efficacy of BTX-A injection in pain relief in IC/BPS patients. Table S9. Meta-regression analysis using complications as moderator to test the efficacy of BTX-A injection in pain relief in IC/BPS patients. Table S10. Meta-regression analysis using concomitant treatment, number of site injections, symptoms duration and complication as moderators to test the efficacy of BTX-A injection in pain relief in GPP patients. Table S11. Meta-regression analysis using symptoms duration and complication as moderators to test the efficacy of BTX-A injection in pain relief in CP/PPS patients. Table S12. Meta-regression analysis using concomitant treatment and number of site injections as moderators to test the efficacy in pain relief of BTX-A injection in the bladder trigone in IC/BPS patients. Table S13. Type and prevalence of adverse events/side effects of BTX-A injections in CPPS for all the 18 included studies. Figure S1. Funnel plot using standard error by Hedges’ of the pooled meta-analysis of 21 selected cohorts coming from 14 included overall studies.
