# Supplementary material for: Botulinum Toxin-A Injection in Chronic Pelvic Pain Syndrome Treatment: A Systematic Review and Pooled Meta-Analysis
Source: Toxins (Basel). 2022 Jan 1;14(1):25. doi: 10.3390/toxins14010025 (PMC8780260; doi:10.3390/toxins14010025)
Supplement: Supplementary file 1 [file toxins-14-00025-s001.zip › supplementary.pdf]

Botulinum Toxin-A Injection in Chronic Pelvic Pain Syndrome Treatment: A Systematic Review and Pooled Meta-Analysis

Andrea Panunzio, Alessandro Tafuri, Giovanni Mazzucato, Clara Cerrato, Rossella Orlando, Vincenzo Pagliarulo, Alessandro Antonelli and Maria Angela Cerruto

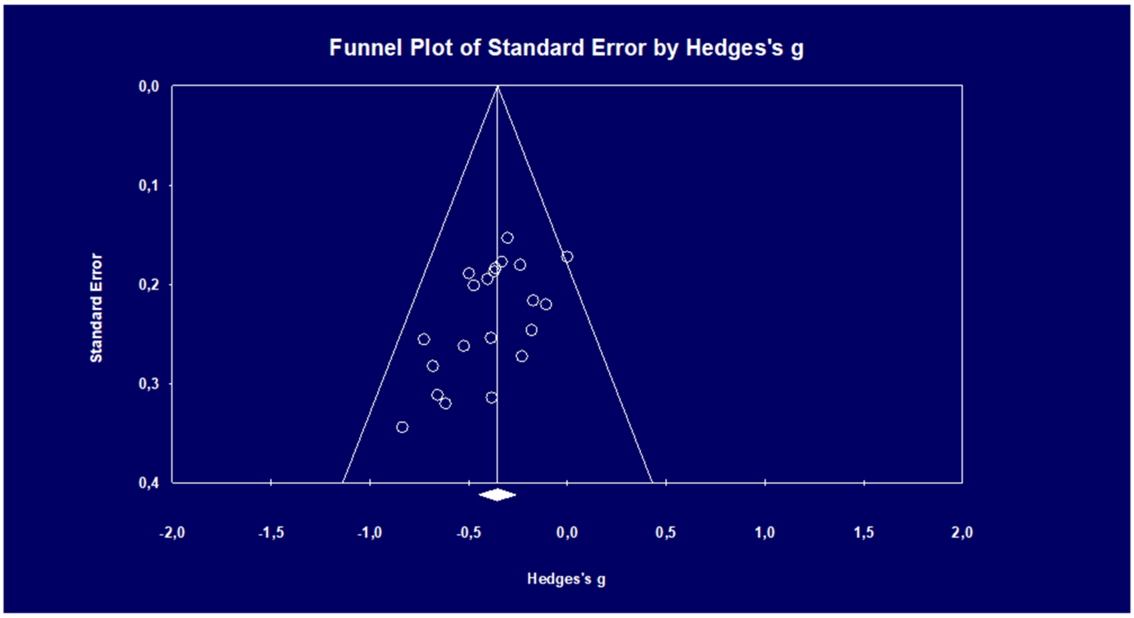

Figure S1. Funnel plot using standard error by Hedges'g of the pooled meta-analysis of 21 selected cohorts coming from 14 included overall studies.
